# Case report: Facilitating right heart recovery after durable LVAD implantation through repair of atrioventricular valves and RVAD implantation using tunneled Dacron grafts

**DOI:** 10.3389/fcvm.2023.1251837

**Published:** 2023-09-07

**Authors:** K. Candis Jones-Ungerleider, Syed Sikandar Raza, Paul C. Tang

**Affiliations:** ^1^ Department of Cardiac Surgery, University of Michigan Frankel Cardiovascular Center, Ann Arbor, MI, United States; ^2^ Department of Cardiovascular Surgery, Mayo Clinic, Rochester, MN, United States

**Keywords:** mitral regurgitation, tricuspid regurgitation, left ventricular assist device, right ventricular assist device, right heart failure, right ventricular dysfunction

## Abstract

Right ventricular assist device (RVAD) weaning is often an important goal for durable left ventricular assist device support. This may be facilitated by mitral and tricuspid repair as well as by minimizing the trauma of RVAD decannulation by using Dacron grafts.

## Graphical Abstract

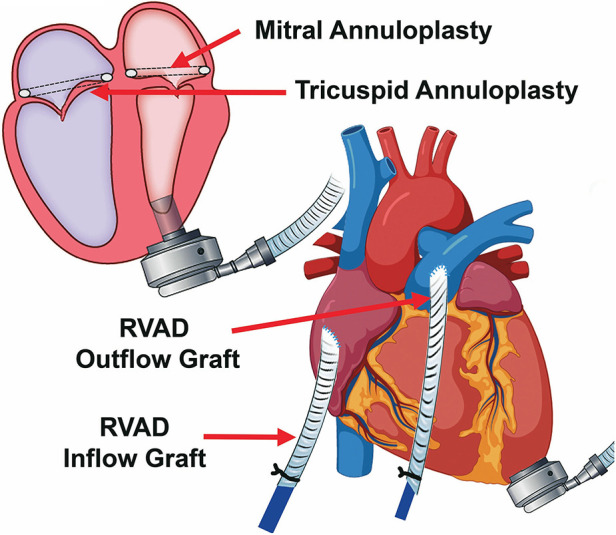
 Concomitant mitral and tricuspid valve repair with biventricular assist device implantation.

## Introduction

Right heart failure after left ventricular assist device (LVAD) implantation leads to major morbidity and mortality (1). Significant residual mitral regurgitation (MR) and tricuspid regurgitation (TR) after LVAD implantation have been found to be associated with right ventricular (RV) failure (2, 3). For patients requiring temporary right ventricular assist device (RVAD) support, concomitant mitral and tricuspid repair may reduce right ventricular afterload and promote unidirectional blood flow to facilitate RVAD weaning and removal. An RVAD strategy that does not require redo-sternotomy for decannulation can reduce surgical trauma to the RV.

## Clinical summary

A 42-year-old man with non-ischemic dilated cardiomyopathy marked by recurrent admissions over the past 3 years presented in cardiogenic shock and with declining renal function, with a MELD score of 24. Echocardiography showed a left ventricular (LV) end-diastolic diameter of 78 mm and ejection fraction of 16% with an enlarged left atrium (LA) of 54 mm. Severe MR with annular dilatation and tethered leaflets was noted, along with severe TR (Supplementary Video S1). Severe RV dysfunction on echocardiogram was consistent with poor hemodynamics, with right atrial pressure of 18 mmHg, pulmonary artery pressure of 52/35 mmHg, cardiac index of 1.47 L/min/m^2^, pulmonary artery pulsatility index of 0.94, central venous pressure to pulmonary capillary wedge pressure ratio of 0.75, and RV stroke work index of 410 cc-mmHg/m^2^/beat. Shock was addressed with percutaneous LVAD (TandemHeart) support for 11 days until end-organ function improved. TR was persistently moderate in severity to severe in severity despite temporary mechanical support.

**Figure 1:**
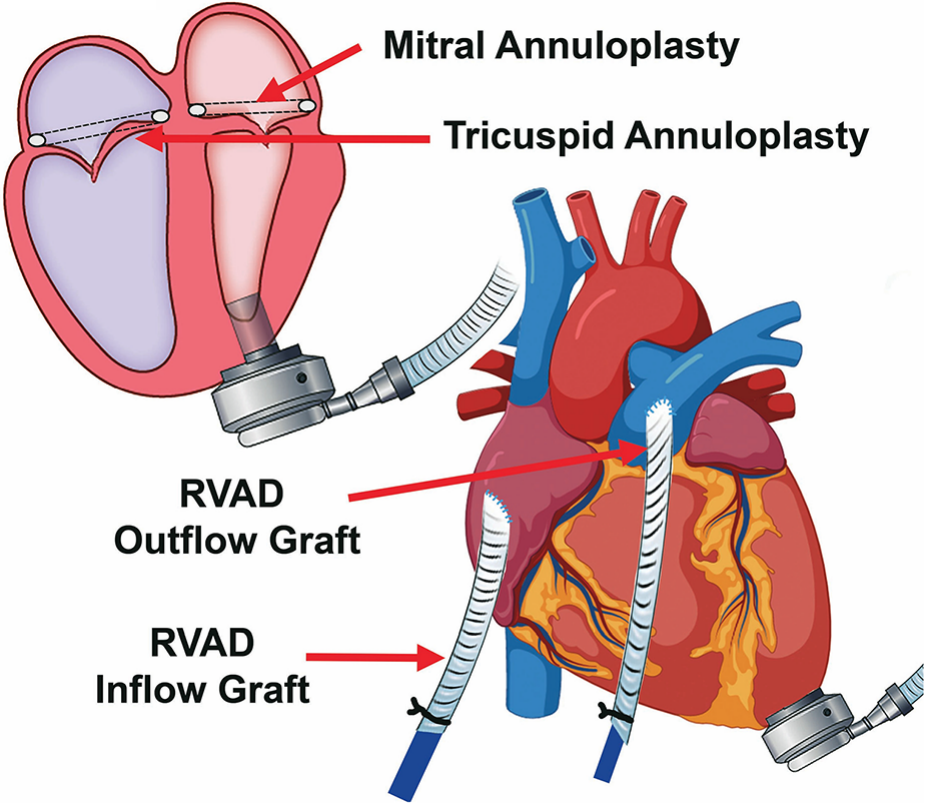
Concomitant mitral and tricuspid valve repair with biventricular assist device implantation.

Biventricular assist device (BiVAD) therapy with beating heart valve repair was then performed as follows. (i) TandemHeart LVAD was weaned. (ii) Cardiopulmonary bypass was achieved with aortic and bicaval cannulation. LV venting via the right superior pulmonary vein, ascending aorta, and LV apex was implemented to minimize the risk of air embolism. (iii) A HeartMate 3 apical cuff was secured to the LV apex, which was then vented. (iv) The interatrial septum over the TandemHeart LA cannula was incised with care to remove thrombus on the cannula to avoid embolization, and the cannula was then removed. (v) The mitral valve was readily visualized via trans-septal incision, due to the large LA and absence of aortic insufficiency (AI). A 32-mm mitral annuloplasty rigid ring was used for annular downsizing. (vi) The septum was closed and a 28-mm tricuspid valve annuloplasty ring was secured to the annulus (Figure 1). (vii) We then removed the LV core, secured the HeartMate 3, and sewed the outflow graft to the ascending aorta.

**Figure 2:**
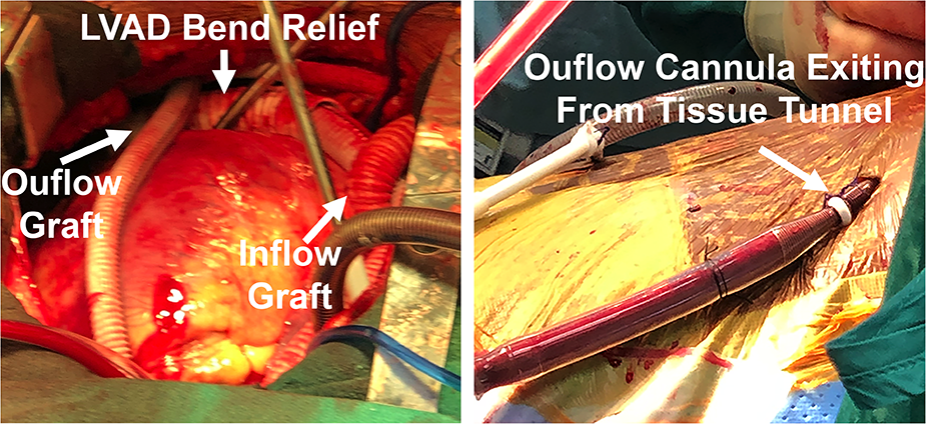
Biventricular device implantation with RVAD cannula placement, including tunneled inflow and outflow grafts to optimize decannulation.

Hemodynamics were poor on attempted bypass wean, and we proceeded with RVAD implant. (i) A Dacron graft (8 mm) was sewn onto the pulmonary artery and tunneled through the skin inferiorly. A 20 Fr arterial cannula was inserted into the graft and secured with umbilical tape and silk ties. (ii) A Dacron graft (12 mm) was anastomosed to the right atrium and tunneled through the skin, and a 28 Fr cannula was then placed and secured (Figure 2). (iii) Cannulas were connected to a CentriMag with flow established at 3.5 L/min (Figure 1). The patient was successfully weaned from bypass on minimal pharmacological support. HeartMate 3 speed was 5,500 rpm with flow at 4.2 L/min. Minimal AI, MR, or TR was noted at completion, and the chest was closed at initial surgery (Supplementary Video S2).

RVAD flow was maintained at 3.5 L/min for the first 5 days for a high level of support. As the patient was extubated, inotropes and pressors were weaned; volume status was reduced with aggressive diuresis in the following days, and the patient’s RVAD flow was also slowly decreased to around 2 L/min for several days. After he had undergone limited rehabilitation with ambulation and had been confirmed to be stable on low-dose epinephrine (0.02 µg/kg/min), the RVAD was then removed in the operating room on postoperative day 15. (i) Inflow and outflow grafts were accessed in the subcutaneous tunnel via overlying incisions. (ii) The RVAD was weaned and cannulas were removed, with the Dacron graft tied off using 3-0 silk sutures and returned to the chest. (iv) Skin and subcutaneous tunnels were closed, with the sites sealed with a wound vac. The patient was discharged on postoperative day 30 without complications. As of the last follow-up at 4 months postoperatively, the patient is alive and doing well without complications or readmissions. He has been ambulating independently and is able to perform activities of daily living without issues while supported with a durable LVAD only.

## Discussion

Right heart failure occurs in 10%–40% of patients following LVAD implantation (4). In particular, for patients who have severe RV dysfunction and are receiving LVADs for destination therapy indications, it is critical to maximize the likelihood of successfully weaning from temporary RVAD support. Our surgical strategies include correction of concomitant valvular lesions to reduce RV afterload and restore forward flow, as well as minimizing surgical trauma to the RV with decannulation. The use of Dacron grafts to minimize surgical trauma associated with RVAD explant has also been described by others (5) and has advantages compared with other techniques requiring redo-sternotomy for RVAD explant (6, 7) or placing limitations on patient ambulation from peripheral ambulation (6, 8).

Our practice is to place a temporary RVAD at the time of initial LVAD implantation in patients with severe RV dysfunction based on preoperative echocardiography and hemodynamics. We feel that this is particularly important in patients with borderline renal dysfunction, where high-dose inotropes and/or hemodynamic instability are associated with a high risk of renal failure and dialysis. We use TandemHeart temporary LVAD in cases of very poor RV function for hemodynamic stabilization as well as to test whether the RV can accommodate the increase in venous return with LV support.

We perform concomitant mitral repair in patients with severe MR, particularly those with large LV dimensions and moderate-to-severe RV dysfunction. Residual MR imposes RV afterload, which is associated with RV failure, renal failure, and higher mortality (9). Although the benefits of tricuspid intervention for moderate-to-severe regurgitation are not well defined, our group performs concomitant tricuspid valve repair for moderate-to-severe regurgitation. We propose a BiVAD support strategy that can promote successful RV recovery in order to facilitate RVAD weaning and decannulation. The twofold strategy consists of (1) concomitant beating-heart biatrioventricular valve repair to reduce valve regurgitation and RV workload, and (2) minimization of RV trauma during decannulation using tunneled Dacron grafts to avoid resternotomy. Furthermore, concomitant correction of valve lesions should be strongly considered if the likelihood of cardiac functional recovery is deemed to be high.

Our group (2, 10) and others (9) have also shown that residual MR after LVAD can contribute significantly to RV afterload, and establishment of mitral competence would be expected to reduce late right heart failure and readmissions. Conversely, the rationale for tricuspid valve repair is to promote right-sided forward flow and reduce the physiological impact of right heart dysfunction (11, 12). Indeed, both the 2019 EACTS Expert Consensus on long-term mechanical circulatory support (13) and the 2023 International Society for Heart and Lung Transplantation Guidelines for Mechanical Circulatory Support (14) state that establishment of mitral competence for pre-LVAD severe MR and tricuspid repair for moderate or severe TR either can be considered or are recommended.

Potential graft infection is an important consideration for this technique. To minimize this risk, we bathe the graft in rifampin, make sure that there is a long subcutaneous tunnel, and also excise the distal ends of the Dacron graft at the time of RVAD explant. There is also a theoretical possibility of pulmonary embolism from thrombus within the graft, but we have not encountered this issue thus far. The pathophysiology of and optimal therapeutic approaches to RV failure in the LVAD setting merit further study and standardization.

## Data Availability

The raw data supporting the conclusions of this article will be made available by the authors, without undue reservation.

## Supplementary material

Video 1 Preoperative transesophageal echocardiogram showing significant tricuspid and mitral regurgitation, trace aortic insufficiency, and severe right ventricular dysfunction. Preoperative TandemHeart device cannula is seen traversing the interatrial septum.

Video 2 Postoperative transesophageal echocardiogram showing trace residual tricuspid regurgitation, no mitral regurgitation, and baseline aortic sufficiency. Severe right ventricular dysfunction necessitated RVAD support. The LVAD inflow is well aligned with the mitral valve.
